# Suffering and loss in Lewy body dementia: Applying a palliative care lens to a longitudinal narrative study

**DOI:** 10.1017/S1478951524001962

**Published:** 2025-06-20

**Authors:** Allison Bentley, Yakubu Salifu, Catherine Walshe

**Affiliations:** 1 Department of Public Health and Primary Care, University of Cambridge, Cambridge, UK; 2 Cambridgeshire and Peterborough NHS Foundation Trust, Windsor Research Unit, Fulbourn Hospital, Cambridge, UK; 3 International Observatory on End of Life Care, Division of Health Research, Faculty of Health and Medicine, Health Innovation One, Lancaster University, Lancaster, UK

**Keywords:** Lewy body dementia, palliative care, Parkinson’s, narrative, longitudinal, dyadic, qualitative, caregiving

## Abstract

**Objectives:**

This study aims to explore the everyday experiences of people living with Lewy body dementia and their families, to deepen understanding of their care needs. Lewy body dementia is a neurodegenerative condition associated with shorter life-expectancy and poorer quality of life than other forms of dementia. Cognitive fluctuations, visual hallucinations, falls, and motor features of Parkinsonism gives rise to complex and debilitating symptoms. Other prominent features include behavioral and emotional problems, rapid eye movement sleep disorder, and autonomic dysfunction. Improving palliative care for people with dementia continues to be an international priority; however, little is known as to how a palliative care approach could support people living with Lewy body dementia and their families.

**Methods:**

Drawing on narrative theory of self and personhood, a qualitative, longitudinal narrative approach provided unique insights into 5 couples’ experiences of living with Lewy body dementia. Analysis was conducted using Murray’s levels of narrative analysis in health psychology to explore stories at the personal, interpersonal, positional, and societal level.

**Results:**

Participants with Lewy body dementia described losses associated with communication, continence, and energy leading to a progressive loss of independence. For their family caregivers a loss of companionship was particularly salient. These losses, compounded by a perceived lack of clinical support, resulted in suffering both for the person with Lewy body dementia, and for those close to them.

**Significance of results:**

There has been a societal and political shift to move beyond loss in dementia, to a focus on abilities and living well. However, acknowledging loss, while supporting symptom management is an important aspect of Lewy body dementia care. Providing person-centered, palliative supportive care throughout the disease trajectory could reduce suffering and enhance well-being.

## Introduction

Multiple complex symptoms and a progressive course of functional decline have resulted in a reframing of dementia as a “life-limiting” illness that requires palliative care (Fox et al. 2018). Policy and research around palliative care in dementia has mainly focused on the final stages of life (Mataqi and Aslanpour 2020). In this context people with dementia are often considered as a homogenous group, eliding the variability between different types of dementia. This variability must be considered if more nuanced understandings of the way people live and die with dementia are to develop.

One form of dementia with particular psychosocial and physical challenges is Lewy body dementia. Lewy body dementia is an umbrella term that includes both Parkinson’s disease dementia and dementia with Lewy bodies. Lewy body dementia differs from other forms of dementia both in terms of its complex and challenging symptoms and its association with a shorter life-expectancy (Liang et al. 2021; Mueller et al. 2019; Price et al. 2017; Wu et al. 2018). People with Lewy body dementia often live with an array of symptoms such as cognitive fluctuations, visual hallucinations, falls, and motor features of Parkinsonism. Other prominent features include recurrent behavioral and emotional problems, rapid eye movement sleep behavior disorder, and autonomic dysfunction (Dubois et al. 2007; McKeith et al. 2017). With no specific disease-modification therapies, clinicians focus on support, aiming to mitigate the complex combination of cognitive, motor, autonomic, and psychiatric symptoms encountered. However, treatment of 1 symptom can exacerbate another which makes clinical management difficult (Taylor et al. 2020), complicating care at the end of life (Armstrong et al. 2019a). Because of these differences it is argued that people living and dying with Lewy body dementia should be considered as a distinct group with regard to their palliative care needs.

Current palliative care research about Lewy body dementia is mainly focused on end-of-life care with bereaved caregivers. Perceived barriers to quality end-of-life care include: lack of physician knowledge resulting in prescribing errors; poor communication and coordination among professionals; and difficulty accessing resources due to behavioral challenges (Armstrong et al. 2019a, 2020, 2019b). While carers experiences have been documented, the voices of those living with Lewy body dementia are largely unheard (Bentley et al. 2021a; Larsson et al. 2019). This research aims to address this by drawing on patients’ and their families’ accounts of the experience of living with Lewy body dementia. A narrative approach allowed people time and space to curate their experiences and present the stories that matter to them, helping us gain a deeper understanding of what their lives were like. To further explore the complexity of living and dying with Lewy body dementia a palliative care lens was applied to the stories, with a focus on loss and suffering.

## Methods

Narrative theory of self and personhood was drawn on to consider that humans understand their world through stories (or narratives), and that knowledge and reality are socially produced. A qualitative design used narrative inquiry to gather and analyze stories over time (Riessman 2008). A patient and public involvement group helped devise and piloted open-ended questions to elicit stories, with the aim to answer the following research questions:

- What are the everyday life experiences of people with Lewy body dementia?
- How do family/informal carers describe the experience of living with someone who has Lewy body dementia?
- How do these stories and experiences relate to palliative care?

### Setting

A community-based study was conducted in eastern England in the United Kingdom (UK).

### Population and sampling

Dyads representing people with a diagnosis of Lewy body dementia and a close family member were recruited to the study using a convenience sampling strategy. The inclusion and exclusion criteria were applied as per Box 1.

Box 1. Inclusion and exclusion criteria

**Inclusion criteria**

***Person with Lewy body dementia*:**

- Diagnosis of either dementia with Lewy bodies (McKeith et al. 2017) or Parkinson’s disease dementia (Dubois et al. 2007) as determined by clinician or participants.
- apacity to give informed consent within the context of an interview.
- Verbal skills sufficient to engage in an interview in English, or able to take part in an assisted interview using storyboarding techniques.
- Close family member/informal carer willing to be involved.

***Family member/informal carer*:**

- Living with, or close involvement with, family/friend with Lewy body dementia.
- Have the capacity to consent on their own behalf.
- English language skills sufficient to engage in an interview.
- Informal caregivers with regular input: spouse, partner or an adult descendant providing care without payment (Tokovska et al. 2022).

**Exclusion criteria**

***Person with Lewy body dementia*:**

- Diagnosis not confirmed, as assessed by clinician, researcher, or participant.
- Lack capacity to consent to participate in the research, as assessed by clinician or researcher.

***Family member/informal carer*:**

- Unable to participate in a qualitative interview using English, as assessed by clinician or researcher.
- Unable to give informed consent within the context of the research, as assessed by researcher.

No apriori sample size was determined, but the number of participants determined using the concept of information power. The aim was to obtain richness of data by focusing on the areas of sample specificity, quality of dialogue, and analysis strategy (Bradbury-Jones et al. 2017; Malterud et al. 2016). The sample size was deemed sufficient when richness of data was sufficient to address the research questions. This was strengthened by use of longitudinal data methods, and the layered analytical process.

## Ethics

Heath Research Authority (HRA) ethical approval was received from an National Health Service (NHS) Social Care Research Ethics Committee (18/IEC08/0035) on 30.11.2018. This facilitated academic approval from Lancaster University (FHMREC). AB, as a nurse researcher, had specific training and experience in assessing capacity and gaining informed consent from people with dementia. Process-ongoing consent was in place, including checking at the start of each interview if the participants were able and willing to continue (McKeown et al. 2009).

### Recruitment

Participants were approached and recruited from a range of sources via voluntary and statutory service sectors. Statutory health and social care services included memory clinics, community teams (combined mental health & primary care multidisciplinary teams), and the psychiatry liaison service within the local NHS trust. Within the NHS trusts staff providing an introductory letter and participant information sheet to eligible participants. This included an “opt in” reply form with stamped, addressed envelope, and the option to telephone or email the researcher. In addition, potential participants could contact the researcher via a poster placed National Institute for Health Research Join dementia research website, the Lewy Body Society.

### Data collection

Three data collection methods were applied to ensure rich data: longitudinal interviews, the option of interviews together with family members, and life story work to support participants living with Lewy body dementia. The aim was to support person-centered interviews, and assist as a communication method for participants with dementia to share their stories and express themselves (Cooney and O’Shea 2019; Gridley et al. 2020; Samsi and Manthorpe 2020). Five married couples completed longitudinal interviews (3 per couple) over a 6-month period in their own homes. Interviews were conducted between July 2019 and February 2020 by AB, an experienced female qualitative researcher with no prior relationship to participants. Interviews were audio-recorded, transcribed verbatim, checked, anonymized, names were replaced with pseudonyms. Transcripts were then uploaded to ATLAS software to facilitate data management. In addition a research diary was used to make field notes as soon as possible following the interviews. Participants were offered a copy of the transcripts for comments or corrections, of which all declined. Credibility and legitimacy was addressed by gathering data at different time points from the same participants, and patient and public feedback on the findings.

### Patient and public involvement

In line with recommendations, and to aid trustworthiness, a patient and public involvement group was created for the study (Burton et al. 2019; Gove et al. 2018). The group consisted of 3 current spousal caregivers, 1 person living with Lewy body dementia, and 1 adult daughter. The group said they don’t view the condition as life-limiting and when they think of palliative care they think of cancer or hospices. They said “*Focus on day-to-day living instead. And talk to people at different time points as their experiences will change*” (Carer: daughter). An interview guide was subsequently developed with the group to identify 5–7 broad, open questions (Box 2). The group also provided feedback on the findings at the end of the study.

Box 2. Interview topic guide

**Person with Lewy body dementia:**

1. I want to find out what it’s like to live with Lewy body dementia. I know it might not be easy to describe. To get us going – if I ask you what it’s like to live with Lewy body dementia, what’s the first thing that comes into your mind?
2. What else comes to mind?
3. What are the main difficulties for you of living well?
4. What gets in the way of doing things you want to do?
5. What helps?
6. Tell me about something you enjoy.
7. Thinking about a nice day out you have had, or holiday –
  - What makes it good?
  - What could have made it better?

**Spousal caregiver:**

1. If I were to ask you what it’s like to live with someone who has Lewy body, what’s the first thing that comes into your mind?
2. What is a typical day like for you and your husband/wife/partner?
3. How much freedom do you have to pursue your own activities and interests?
4. What gets in the way of you doing things you would like to do yourself?
  - The 2 of you together?
5. What/who helps make life easier?
6. How have things changed over time?

### Researcher positioning

All authors have a background in nursing and palliative care academia with AB having 10 years’ experience of working clinically and academically within the field of Lewy body dementia. This research is therefore approached from a variety of stances. These include the medical model of dementia as a progressive neurodegenerative disease, specialist palliative care, and a community nursing perspective.

## Data analysis

Murray’s (2000) levels of narrative analysis in health psychology was applied to the narrative data collected. This was chosen as it provides a multidimensional approach to health and social illness narratives whilst acknowledging that individual experience is centralized within the interconnected social and cultural context (Murray 2000; Murray 2015). Murray’s analytical framework enabled stories to be analyzed at the personal, interpersonal, positional, and societal level. Supplementary Table S1 provides more detail of Murray’s (2000) 4 levels of narrative analysis, with related analytical questions applied to the data.

Initial listening and reading of transcripts resulted in the study data being organized into a short descriptive profile for each couple. This aids in the identification of stories and content of the longitudinal interviews (Murray and Sools 2015). The intension was to apply a narrative analysis to retain the individual stories, rather than fragment into categories and codes. At the individual personal level of analysis the setting, plot, characters, and main event of each storyline were identified by AB, and a title was applied to capture the main focus of the story (Murray and Sools 2015). Underlying narratives were identified from within each story and a time ordered sequential matrix was then created to explore if stories reoccur, or change over the 3 interviews (Bentley et al. 2021b; Grossoehme and Lipstein 2016). During the analysis phase monthly discussions took place with 2 additional researchers (CW and YS) on the plausibility of the interpretations of the stories and underlying narratives identified. This involved exploring both individual stories as well as the stories that emerged as the couples interacted with each other. We then considered if couples had shared, or differing experiences of the same stories. The final stage involved connecting and comparing stories across couples to identify the overarching narrative. A more in-depth application of Murray’s levels of narrative analysis to the data is published elsewhere (Bentley et al. 2021b). Supplementary table S2 provides detail of the narrative analysis process and findings.

## Findings

Ten participants (5 couples) were recruited and completed the study. Time spent with each couple over their 3 interviews ranged from 1 hour 21 mins to 3 hours 37 minutes, with a mean time of 2 hours 43 mins. Participant characteristics are presented in Table 1.

**Table 1.** Participant characteristics

Participants	Person living with dementia *N* = 5	Spousal caregiver *N* = 5
**Age (years)**		
65–69	1	2
70–74	4	2
75–79		1
**Gender**		
Male	3	2
Female	2	3
**Years diagnosed**		
1–5	3	
6–10	2	
**Ethnicity**		
White British	5	5
**Diagnosis**		
**DLB**	4	
**PDD/DLB** unclear	1	

Dementia with Lewy bodies (DLB), Parkinson’s disease dementia (PDD).

Pseudonyms have been used to protect anonymity, with names in **bold** representing the person living with Lewy body dementia. The spousal couples include **Patrick** and Sue; **Kath** and Ken; **Joan** and Peter; **Jack** and Linda; **Doug** and Gayle. The term “*spousal caregiver*” is used when discussing the spouse who does not have Lewy body dementia. **Jack** and **Patrick** had been recently diagnosed with Lewy body dementia, **Kath** and **Doug** had been living with the condition for several years. **Doug** and **Joan** both died a few months after the final interviews.

**Figure 1.**
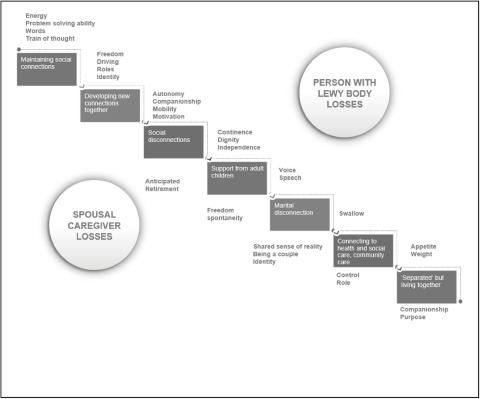
Social connectedness and associated losses – person with Lewy body dementia (represented in upper-right section) and spouse (lower-left section).

The findings identified that the overarching narrative for the couples, over time was social connectedness. Participant stories show that whilst new connections could be made, there was an overwhelming sense of individual, and shared loss. Although the couples made some new connections, the overall trend was a deterioration in ability to remain socially active and an overwhelming sense of individual, and shared loss. The longitudinal narrative findings are presented as a stepwise decline, with associated losses (Fig. 1). As Gayle said “*I don’t know where we’re at in the progression of the Lewy body, you know it fluctuates but you’re going down this … graph, but it’s only going down one direction.*”

Data is presented focusing on stories of loss which had the greatest impact on remaining socially connected. Communication and loss of voice; reduced autonomy; loss of energy and motivation; loss of companionship and being a couple. Supplementary Table S3 provides relevant quotes representative of the language of loss.

### Communication and loss of voice

Cognitive and physical Parkinsonian changes played a significant part in participants’ difficulties in communicating with each other as a couple, with the wider world, and with health care professionals. Cognitive changes affected communication early in the disease process. Participants described slowness in retrieving words, loss of words, problems with word linking, and poor memory. **Jack** described his experience of retrieval difficulties:

Jack: *There was one other thing I was going to say to you, which I can’t remember, I really do suffer from a lousy memory …. I’m sure that if I could just bridge my mind it would come up with all interesting things. But I can’t bridge my mind I’m afraid.*

**Kath** often hesitated, saying, “*I’ve lost my train of thought now.*”

Loss of voice was also evident due to physical Parkinsonian changes which resulted in the voice becoming quieter. This often occurred later, as the condition progressed (Fig. 1). **Patrick** described his experience of voice changes as going “*from being quite high level to um mumble. I lost my voice early on, then it came back. I mean thinking about it, it is down, definitely up and down.*”

Spousal caregivers described how cognitive and physical communication difficulties often left them feeling frustrated and guilty. “*I can’t understand what **Doug’s** saying at all and it gets frustrating for both of us. And sometimes he’s quite clear*” (Gayle).

Couple interactions also highlighted communication difficulties. Sue felt it was partly her fault when communication between them wasn’t going well. “*I suppose it is down to me, I was cross and I was frustrated and of course I suppose that reflects [on me].*” A guilty feeling was similarly expressed by Peter, when he reflected “*it might be my fault I don’t know, but there’s not much conversation going on at all, it’s me that ends up doing the talking.*” Challenges occurred when acknowledging and talking about the condition. Peter felt it was unkind to talk to his wife, Joan, about her diagnosis. He explained this in terms of his wife’s mind “going wrong”:

Peter: *The way our minds work and when they go wrong you can’t talk like that and we’ve really never talked about really have we **Joan** your condition as such, it’s very difficult to say if I start trying to explain what I think is happening, that’s not really very kind and um …*

**Jack** and Linda avoided the subject because both found it too distressing:

Linda: *We don’t talk about it.*

Jack: *Talking about it is the worst thing going ….* [Living with Lewy body dementia is] *‘very off putting, it’s worrying and you wish it would go away but it ain’t going to. So you know, I’m distressed today’.*

The stories involving loss of voice, difficulty being heard and connecting to others illustrated the complex changing, and differing communication needs over time.

### Loss of energy and motivation

People with Lewy body dementia in this study attributed their reduced social interaction to excessive tiredness, fatigue, and mental slowing down:

Sue: *We have actually just been down to see a friend of Patrick’s … we haven’t said this, but I realised you were, you know he was tired. Both sort of physically and mentally so um we came back.*

Patrick: *It does take energy out of me, but I think I, I could do two hard days.*

Some couples disagreed about how best to respond to this fatigue. **Kath** was determined to “*fight*” her condition, although tiredness and fatigue became a major difficulty. Ken implies that pacing herself would be better, perhaps indicating a tension between unwisely doing too much and consequences of doing too little.

“***Kath*** *just won’t give in to anything, and then next day you know she’s tired and and wants to spend the day in bed so she’s her own worst enemy in that respect.*” *Later in the interview*, **Kath** admits “*I am tired. That’s how I feel, um at the moment.*”

**Jack** and Linda’s disagreements mirrors **Kath** and Ken’s. **Jack** admits he’s “*not always motivated*” and has to pace himself. He has started to accept that he can’t do as much, saying “*there are limits and I think I’ve accepted that*”; meanwhile Linda has struggled to adapt to these limits, suggesting that is good to keep “*doing.*”

Jack: *I do feel that. … it’s good for me to take things you know not too enthusiastically.* Linda: *But we do things, it’s better if you’re doing you know.*

### Loss of autonomy

Participants living with Lewy body dementia emphasized through stories their loss of identity, freedom, and independence. For the spousal caregiver, they expressed sadness at their loss of spontaneity, control, and roles (Fig. 1). Giving up driving created an immediate physical distancing from social relationships or usual hobbies for the person with Lewy body dementia. In addition to these practical effects male participants acknowledged that driving was an integral part of themselves. **Patrick** described his sense of reduced agency as being “*constricted.*” “*I’m not locked up but there is a certain constrictment, getting around.*” **Doug** also expressed sadness when talking about giving up driving. “*Sadly I can’t do it anymore.*”

**Joan** used narratives of imprisonment and not being “released” to describe her loss of mobility and agency:

Joan: *I was capable of being able to walk, whereas now I’m mostly being prisoner here.*

Interviewer: *Prisoner here, you mean in bed?*

Joan: *Mmm. I have all my um meals … It’s frustrating … Unfortunately she [the occupational therapist] concluded that I wasn’t ready to be released.*

**Kath** difficult to reconcile their loss of independence throughout the course of the interviews. Initially **Kath** fought the changes: “*I do fight it, very much so, ‘cos I won’t let it get hold of me.*” However, falls and subsequent hospital admissions led to a step down in her mental and physical function, eventually forced her to face the problem.

Kath: *Um mobility at the moment is quite frustrating for me now. I do have a walker. My daughter got it for me and I was a bit upset, you know, and I wouldn’t even think about it. But now I know myself mobility is getting worse, so you know … I cried ….*

Loss of freedom and spontaneity were exacerbated by continence issues. **Patrick** had urinary and fecal incontinence and Sue described how the loss of continence was affecting **Patrick’s** desire to leave the house.

Sue: *I find it frustrating, I just said to him I want his quality of life what’s best for him, I don’t mind clearing up, I really don’t mind, but it doesn’t make him happy and very comfortable. This morning the same thing, do you want to come shopping, ‘cos we usually go together, and it’s to do with if he makes a mess and I don’t want that to happen do you know what I mean?*

Sue, Gayle, and Linda described how both bladder and bowel issues affected their ability to go out together with their husbands. As Gayle recalled “*I might sound cowardly but I’m frightened, I’d be worried that we’d have an incident like we had on the motorway last Summer.*” By the final interview Gayle said she was severely restricted by **Doug’s** continence issues, as very few sitters would be able to cope if she went out. Linda described how side effects of **Jack’s** medication restricted his activities:

*He struggled ‘cos they’ve changed his medication, he’s on medication for depression, anxiety, which gave him diarrhoea, he couldn’t go out anywhere* (Linda).

As participants adjusted to progressive psychosocial and physical losses, they encountered a wider range of community services. For the spousal caregivers this period highlighted their changing roles from partner to carer and advocate, with loss of spontaneity, control, role, and purpose (Fig. 1). As **Joan’s** condition deteriorated due to another fall and pneumonia, she was deemed in need of palliative care. Carers were provided by NHS Continuing Healthcare 4 times a day. Initially Peter “*felt quite redundant*” from his caring role when the NHS care team came. He found “*not really doing anything with any particular purpose … except to keep the day going*” very difficult, but “*I know I’ve got to look after medication*” and “*learn the best way to help **Joan** eat.*” Peter felt they were both well supported from the hospice, which helped him to adjust. **Joan** attended day therapy when she was able, and Peter accessed some counselling sessions – “*they’ve supported me as well.*” This positive account, however, should be read alongside Joan perception that she was imprisoned, waiting for a therapist to “*release*” her. The challenge for care providers is to offer adequate support while still maximizing the patient’s sense of autonomy.

As **Doug’s** condition progressed, Gayle found it more difficult to maintain her hobbies and interests due to lack of sitters and respite care: “*there’s not the services there, that’s what I find so hard. I just can’t do all the things I like to do.*” She has managed to find a local home help for 3 days a week, “*largely funded by the attendance allowance, she helps [**Doug**] with his shower on the day she comes in*,” but was finding it difficult to cope. At the end of the final interview Gayle says “*we’re beginning to look at care homes*.” The family felt it was important that the decision to move to a care home was made “*while **Doug’s** able, to get around and look at places, so we feel he’s had a say in it*.”

**Doug’s** family struggled to get the help and support he and they needed. The findings also highlighted that access to end of life care services varies, something that is commonly reported in relation to all dementias.

### Loss of companionship and being a couple

For the caregivers, behavioral and psychiatric symptoms affected relationships with their spouse, and contributed to the loss of companionship. Peter talked a lot about the loss of his relationship, eventually saying “*I suppose the other things I miss most is just the companionship really … Probably sadly the most I ever talk to **Joan** about are some of those things which aren’t real anyway …*.” Delusions and hallucinations added to the loss of a shared sense of reality. Sue found it particularly distressing when Patrick mistakes her identity:

Sue: *[Sometimes] I’m a Cook. I was the gardener yesterday … At the beginning it was really quite distressing, um but er you know I will sit and he’ll suddenly say where’s the nice lady that made the tea [laughter].*

Patrick: *I can’t explain that.*

Personality changes were a further source of sadness to spousal caregivers. Sue talked of the loss of the person she knew. As she explained; “***Patrick’s*** *personality is there but it um you know it’s I want to say it’s changed*.” His lack of insight was particularly distressing for her:

Sue: *… I mean it’s the most horrendous um I was going to say medical condition. My mother had cancer, Patrick’s mum had dementia and we both decided that having cancer was better than having dementia. Although my mother knew she was very ill, she knew what was happening, whereas sometimes he doesn’t*.

Paradoxically as spousal caregivers were obliged by the illness to become physically closer to their partner with Lewy body dementia, they felt further away emotionally as their partner’s psychiatric symptoms worsened and their personality changed.

## Discussion

This study found that people’s ability to have a sense of agency may be constrained by both the neuropathology, symptoms of dementia, by the external actions of others and perceived lack of health and social care services. It is said people with Lewy body dementia may lose independence earlier than other forms of dementia (Galvin et al. 2010b), and that giving up driving and reduced mobility in particular diminished independence and self-sufficiency (Larsson et al. 2019). Findings highlighted that loss of energy and motivation, communication challenges; reduced autonomy due to continence and falls; and loss of companionship affected couples’ ability to live well and remain socially active.

Apathy is a commonly reported neuropsychiatric symptom of Lewy body dementia. It can result in of loss of motivation, reduced social interests, emotional blunting, and decline in self-starting behavior (Robert et al. 2009). It is said to be associated with low mood and depression, faster cognitive decline, and early nursing home admission (Breitve et al. 2018). Tiredness, as described by the participants in this study, may precede a feeling of lack of motivation and apathy (Breitve et al. 2018; Drijgers et al. 2010; Eglit et al. 2021; Liu et al. 2019). This is important because apathy and depression make it more difficult for people to participate in therapeutic interventions or group support (McCormick et al. 2019). The presence of apathy adds to caregiver burden, as it leads to a decrease in engaging in social activities which can lead to a reduction in quality of life (Terum et al. 2019; van de Beek et al. 2019; Zweig and Galvin 2014). The spousal caregivers expressed extreme tiredness due to their additional caring roles, lack of sleep, and respite care. Similar experiences were also reported by other family caregivers of people with Lewy body dementia. It was found dealing with mood swings, sleep issues and apathy concerning, and gave rise to disturbed sleep for the caregivers (Stacy et al. 2021).

Changes in communication and ability to interact played a significant part in the loss of connections for people with Lewy body dementia in this study, and are associated with a decreased desire to be active outside the home (Larsson et al. 2019). A meta-synthesis of experiences of living with all types of dementia, found people frequently reported “difficulty engaging and sustaining meaningful, coherent conversations with others,” which resulted in a gradual withdrawal of social interactions (Górska et al. 2017,p. 189). However, the findings from this study highlighted that given the right environment (time, space, and familiarity), verbal and expressive communication, as well as insight, may be well preserved in some people with advancing stages of Lewy body dementia. For 2 participants, the ability to verbally interact, and to express insight and emotions was evident up until the end-of-life stage. Zweig and Galvin (2014) suggested people with Lewy body dementia retain insight into their condition for longer than people with Alzheimer’s dementia, and Larsson et al. (2019) found that despite altered verbal fluency, people with dementia with Lewy bodies demonstrated insight into the complexities of their own cognition, emotions, and symptoms.

The need for independence, autonomy, and a sense of agency for people living with Lewy body dementia is reflected in the general dementia literature (Nimmons et al. 2023). However, bodily changes, particularly around incontinence, and reduced mobility due to falls were key influences in reducing social interactions. Both general dementia literature and Lewy body dementia studies highlight continence issues for the person with dementia had profound effects on quality of life, with the main challenges being the availability of support, financial cost, as well as social, relational, and emotional issues (Allan et al. 2006; Gove et al. 2017; Lee et al. 2018; Stacy et al. 2021). Incontinence is also associated with increased risk of falling in both the general and older populations (Moon et al. 2021). Falls are common in patients with Lewy body dementia and form part of dementia with Lewy body diagnostic criteria (McKeith et al. 2017). A retrospective study of causes and outcomes of hospitalizations in Lewy body dementia found 24% of admission reason was as a result of falls (Spears et al. 2019). The fear of falling and being injured are also contributing factors to isolation and reduced quality of life (Larsson et al. 2019).

Caregivers described how the changes to their spouses’ personalities, hallucinations, and mistaken identity resulted in loss of companionship for them. Behavioral and psychiatric symptoms, particularly common in Lewy body dementia, including hallucinations, delusions, and Capgras syndrome are frequently reported as particularly stressful for caregivers (Galvin et al. 2010a; Leggett et al. 2011; Londos 2018; Park et al. 2018; Shin et al. 2012; Svendsboe et al. 2017; Thaipisuttikul et al. 2013). These symptoms are associated with higher levels of burden, emotional distress, with increased pre-loss grief in caregivers (Park and Galvin 2021). From a theoretical perspective findings in the current study are consistent with the literature on dementia grief theory, and pre-loss grief for Lewy body dementia caregivers (Park and Galvin 2021), and carers more generally (Blandin and Pepin 2017; Chan et al. 2013). Dementia grief is distinguished from anticipatory grief associated with other conditions by disruptions in communication and impairments in awareness that occur even early on in the disease (Blandin and Pepin 2017). These early disruptions are particularly problematic for people living with Lewy body dementia, giving rise to difficulty adjusting to or accepting often-irresolvable losses and may lead to an overwhelming sense of anxiety and suffering. To address these problems there needs to be a “social and emotional space in which people with dementia are supported to live not with loss, but “*in* loss” (Chan et al. 2013, p. 335). In addition, due to the shorter duration of the disease there is typically less time for carers of those with Lewy body dementia to process this grief compared to other forms of dementia, such as Alzheimer’s (Rigby et al. 2021). So whereas the final days of life require similar care regardless of the type of dementia (Armstrong et al. 2019a), people with Lewy body dementia have a particular need for palliative care in the earlier stages of their illness. This is because palliative care support can offer both acknowledgement of loss and suffering and while giving prominence to individual capabilities (Bartlett et al. 2017; Öhlén et al. 2017).

### Strengths and limitations of the study

A strength of the study was the longitudinal, dyadic approach to collecting narratives. This approach made it possible to explore how couples developed a shared narrative that evolved across successive interviews. The couple’s intimate knowledge of each other provided a common reflective space that produced rich data, both in terms of expanding and corroborating a story. The couples opened up about how they found it difficult talking about their situation or the condition. Although there were examples when the interaction had the effect of silencing an individual’s account such as when 1 partner finished the other’s sentences, this diminished over time. Our data surfaced participants’ feelings of loss and distress and revealed how their perception highlighted unique features of Lewy body dementia.

The study was limited by the mainly rural geographical area where participants were recruited from, and by the relatively narrow demographic of participants. These limitations reduce the transferability of our findings to people living alone, in urban areas, or from ethnically diverse backgrounds may be limited. However, the anonymized findings were shared with family caregivers from a different, urban location, who reported similar experiences. It is also important to acknowledge that this study represents the authors’ interpretation of the participants’ stories, and that the stories themselves are co-constructed. Therefore, the findings may differ if approached by researchers from other backgrounds and specialties.

## Conclusion

Living with Lewy body dementia frequently involves loss and suffering. Loss of energy and motivation, communication challenges, reduced autonomy due to continence and falls, and loss of companionship reduced people’s ability to live well. People living with Lewy body dementia would benefit from person-centered support to help them deal with complex symptoms and associated losses. In addition, caregivers require support with pre-loss grief to acknowledge and address multiple losses and suffering to help people remain socially connected. At an individual level a holistic, palliative care approach is needed to support patients and families facing the multiple progressive losses that typify this condition.

### What is already known about the topic

- Lewy body dementia is a condition with complex physical, cognitive, and behavioral symptoms. It is associated with a shorter life-expectancy and poorer quality of life compared to other forms of dementia.
- These symptoms pose particular challenges for family caregivers, increasing their vulnerability to pre-loss grief.
- As in other types of dementia, patients and their families often have unmet palliative care needs.

### What this study adds

- The voices of people living with Lewy body dementia are rarely heard or researched. This study addresses this important knowledge gap, and highlights the multiple losses that patients and family caregivers experience.

### How this study might affect research, policy, or practice

- Understanding the experience of those living with Lewy body dementia is an essential step towards improving their care. Supporting patients and their families to discuss and grieve for multiple progressive losses is important given the inexorable deterioration and challenging symptoms.
- This study highlights the need to develop a consistent, interdisciplinary care pathway with a palliative, person-centered approach for people with Lewy body dementia.

## Supporting information

10.1017/S1478951524001962.sm001 Bentley et al. supplementary material 1 Bentley et al. supplementary material

10.1017/S1478951524001962.sm002 Bentley et al. supplementary material 2 Bentley et al. supplementary material

10.1017/S1478951524001962.sm003 Bentley et al. supplementary material 3 Bentley et al. supplementary material

## Supplementary material

The supplementary material for this article can be found at https://doi.org/10.1017/S1478951524001962.
