# Supplementary material for: Suffering and loss in Lewy body dementia: Applying a palliative care lens to a longitudinal narrative study
Source: Palliat Support Care. 2025 Jun 20;23:e117. doi: 10.1017/S1478951524001962 (PMC13166590; doi:10.1017/S1478951524001962)
Supplement: Bentley et al. supplementary material 1 — Bentley et al. supplementary material [file S1478951524001962sup001.docx]

Supplementary table 1: Murray’s four levels and related analytical questions (adapted from Murray, 2000; Murray and Sools, 2015)

**Bentley A, Salifu Y and Walshe C** (2021b) Applying an Analytical Process to Longitudinal Narrative Interviews With Couples Living and Dying With Lewy Body Dementia. *International Journal of Qualitative Methods* **20**, 160940692110606. https://doi.org/10.1177/16094069211060653.

| Level | Health & illness narratives | Narratives portrayed as: | Analytical considerations/questions |
| --- | --- | --- | --- |
| 1 | Personal | Expressions of the lived experience of the narrator. | What stories are being told?  Which events are noted?  Who are the main characters and sub-characters in the story?  Where/what is the setting (physical or psychosocial) for the story?  Are there divergent perspectives in dyads’ experience of same story?  Which stories reoccur and change over time?  What is the under-lying narrative thread of the stories? |
| 2 | Interpersonal | A dialogue which is ‘co-created’ and structured between participants and interviewer. | In what context does the interview take place?  How do couples’ interactions/relationships influence the storytelling?  How is it determined which stories fade, and which dominate and get told?  Is there an indication that the stories are told with an intention to be read or heard?  How do the participants articulate which are important stories? Are there messages to be conveyed? |
| 3 | Positional | The differences in social position between the narrators and researcher/listener. | Within the interview interaction what roles and social characteristics are known or emerge?  How may the characteristics (gender, age, health status, background, role, etc) of the researcher affect the storyline?  How do the interviewers’ questions, responses, and behaviour influence the story? |
| 4 | Societal/ideological level | Shared stories that are shaped by the social context and ideological assumptions within which we live. | How does the social, cultural, and historical context influence the stories?  What broader societal (or community-based) assumptions (thoughts, practices, language) are evident? |
|  | Connecting the levels | An integration of the 4 different levels of narrative analysis | What is the narrative thread evident at each level?  Is there a connecting, underlying narrative woven through each level?  What new evidence does it offer? |
