# Supplementary material for: Suffering and loss in Lewy body dementia: Applying a palliative care lens to a longitudinal narrative study
Source: Palliat Support Care. 2025 Jun 20;23:e117. doi: 10.1017/S1478951524001962 (PMC13166590; doi:10.1017/S1478951524001962)
Supplement: Bentley et al. supplementary material 2 — Bentley et al. supplementary material [file S1478951524001962sup002.docx]

| Supplementary Table 2. Narrative analysis process and findings table | | | | |
| --- | --- | --- | --- | --- |
| Participants | ‘Small’ stories  (Personal stories) | Underlying narrative | ‘Big’ Story  Narrative threads over time | Overarching narrative |
| 01  **Patrick** and Sue | Getting a diagnosis  Maintaining a social life  Driving  Working life  Friends and family  Pills and potions  Interactions with health and social care  Music  Bowels and bladder  Mistaken identity  Being a collector (life story work) | Piecing together  Struggling to maintain, tiring  Giving up, constricting  Being organised  Supportive ‘back up’  Balancing act/frustrated  Searching for right support, frustration  Reconnecting, coping  Quality of life restricted  Distressing, loss of person  Link to the past, reminiscing | Maintaining social connections  Developing new connections together  Social disconnections  Support from adult children  Marital disconnection    Connecting to health and social care  ‘Separated’ but living together | Social connectedness and loss |
| 02  **Kathleen** and Ken | Getting a diagnosis  Getting out and about (life story work)  Hospital admissions  Family  Medications  Interactions with professionals  Mobility  Swallowing  Toilets/  Bathing and showering  Gardening  Hallucinations | Battle, Frightening  Not giving in, delight when goes for a walk alone, denial  Exhausting, frightening  Supportive, feeling indebted  ‘Wonder drugs’, control  social carer, companionship  Frustrating, keeps pushing, tiring  ‘own worst enemy’  Wary of meals out/socialising  Worry about access  Loss of independence  Retreat from tensions, sanctuary  Learning to manage |  |  |
| 03  **Joan** and Peter | Getting a diagnosis/prognosis  Driving  Hospital admissions  Equipment  Grandchildren (life story work)  Voice changes  Dreams and delusions  Becoming a carer  Medications  Hobbies and interests  Interactions with community services  Relationship changes  Keeping mobile  NHS Continuing Care  Eating and drinking  Palliative care | Uncertainty, stasis  Traumatic, eventual resignation  Speaking out  Confliction, disruption  Miss playing with them, tiring  Difficulty being heard  Unreal conversations, distressing  Learning through experience  Looking things up, responsibility  Becoming dependent, motivation v introjection  Connecting and communicating with rigid systems  Marital disconnection, loss of companionship  Fear of falling, vigilance  Learning the ‘system’  Learning by experience  Supportive services, Decision making responsibility |  |  |
| 04  **Jack** and Linda | Living with a diagnosis  Walking  Family  Groups and interests  Planning and organising  Hallucinations  Medications  Community services  Driving  Working and retirement | Worrying, disconcerting  Important for independence  Providing care, support for carer  Self-sufficiency, companionship  Reduced motivation, tiring  Learning to live with  Struggling with side effects  Discord  Resigned, dependency on spouse  Struggle to adjust |  |  |
| 05  **Doug** and Gayle | Dogs  Music/singing  Groups and activities  Driving  Rural life (Life story work)  Respite care  Therapy support  Friends and family  Eating and drinking  Caring roles  Toileting  Diagnosis/ Prognosis | Companionship, socialising  Voice strengthening  Maintain social interactions  Carers relief when stopped, worrying, frightening  Maintaining links, socialising  Challenging, lack of resources,  Impressed with lateral thinking  Teamwork, sense of humour  Difficult to manage, saliva, cough, embarrassment  Constraining, constricting, strain  Holding on to self-respect.  Uncertain ‘bumpy ride’ |  |  |
