# Supplementary material for: Suffering and loss in Lewy body dementia: Applying a palliative care lens to a longitudinal narrative study
Source: Palliat Support Care. 2025 Jun 20;23:e117. doi: 10.1017/S1478951524001962 (PMC13166590; doi:10.1017/S1478951524001962)
Supplement: Bentley et al. supplementary material 3 — Bentley et al. supplementary material [file S1478951524001962sup003.docx]

Supplementary table 3: The language of loss

| Physical Loss: | Quotes |
| --- | --- |
| Loss of energy/ motivation | Sue: “*We have actually* j*ust been down to see a friend of* ***Patrick’s****….we haven’t said this, but I realised you were, you know he was tired. Both sort of physically and mentally so um we came back.*  **Patrick**: “*It does take energy out of me, but I think I, I could do two hard days.”*  Linda: *“Well motivation’s an issue isn’t it with this dementia isn’t it.* **Jack**: *I’m not always motivated…. there are limits and I think I’ve accepted that”*  **Jack***: “I do feel that.…it’s good for me to take things you know not too enthusiastically.”*  Patrick: *Tired. I was tired this morning and I’m definitely tired now, but I’ve felt tired for.* Sue: *Well we’ve been away as well.*  **Kath:** *I went out with my carer yesterday…I said I’d love to go out, you know. But I was I was tired, I needed to get back if you know what I mean, we had coffee, I said I need to get back now, I felt like I’d had enough.*  Ken: *“Kath just won’t give in to anything, and then next day you know she’s tired and and wants to spend the day in bed so she’s her own worst enemy in that respect but she won’t try and pace herself.”*  **Kath:** *“I am tired. That’s how I feel, um at the moment, um.”*  Linda: “*I couldn’t understand it. I never thought you know it was that [Lewy body], he was just tired,…”*  Sue: “*I said oh I’m Sue, oh yes yes that’s right, so no I’m tired, difficult week.”*  Sue: *“….he often falls asleep once his brother’s been here* *which is fine ‘cos it’s tiring,”*  Gayle: *“Sunday he slept a huge amount but then we had been out quite late the evening before so he was really tired but there’s no real pattern to it, he just nods off.”* |
| Loss of word finding, memory, voice | Patrick: *“It was deemed I think that, the we didn’t need um um, sorry I’m thinking of the wrong words.”*  Patrick: ***“****Not like I’m free like a bird….Um, there the situation I’m in is, can’t think of the word…..”*  Patrick: *“I mean my vocabulary is mixed, it’s gone from being quite high level to um mumble. I lost my voice early on, then it came back. I mean thinking about it it is down, definitely up and down.”*  Gayle: *“Sometimes I can’t understand what Doug’s saying at all and it gets frustrating for both of us. And sometimes he’s quite clear….*  Gayle: “*I suddenly find something you can’t do or can’t find or can’t remember, but you could remember, did know the day before. But on the day in question you might not know it at all and you can’t anticipate, so it tries your patience rather a lot. And other days he’s brighter and chattier and you know, it’s the fluctuation I think makes it challenging”.*  **Jack:** *“There was one other thing I was going to say to you, which I can’t remember, I really do suffer from a lousy memory… . I’m sure that if I could just bridge my mind it would come up with all interesting things. But I can’t bridge my mind I’m afraid.”*  Peter: *“ I find it quite difficult to hear Joan at times, But* ***Joan*** *was never a loud person….you’ve got quieter and quieter haven’t you? Joan: Yes*  **Kath:** “*I’ve lost my train of thought now”*  Sue: “*I suppose it is down to me, I was cross and I was frustrated and of course I suppose that reflects [on me]”.*  Peter: *“it might be my fault I don’t know, but there’s not much conversation going on at all, it’s me that ends up doing the talking”.*  Peter: “*The way* *our minds work and when they go wrong you can’t talk like that and we’ve really never talked about really have we* ***Joan*** *your condition as such, it’s very difficult to say if I start trying to explain what I think is happening, that’s not really very kind and um… .”*  Linda: “*We don’t talk about it.*  Patrick: *Talking about it is the worst thing going….”* |
| Emotional/social loss |  |
| Loss of confidence | Sue: *“he’s not quite as confident and I don’t know whether I’m overhelping him because I don’t want to him to lose his confidence or his independence um but.”*  Gayle: “*The bad winter two years ago he fell over on the ice and um lost his confidence [walking the dogs], he did have a pendant alarm with GPS on it which was good ‘cos, it gave him a bit of independence…”*  Jack: *“Well if it is Lewy whatever it is, then um it’s very off putting, it’s um and it’s worrying and you wish it would go away but it ain’t going to”.* |
| Mutual/ couple loss |  |
| Loss of roles/identity/purpose | **Jack:** *“I mean like most folk in my position you know they’re very dedicated to their jobs aren’t they, you know but er I need to give up the job, I mean that’s something else major that I had to give up, June didn’t have to but I had to, I just realised that I couldn’t do what I was trying to do.”*  Linda: *“I think he was struggling because he changed his role, normally it wouldn’t bother him but he just struggled and I thought this is really funny, you know he can’t get a grip on this new [role] but he really seemed to be struggling and I thought why don’t you retire….I couldn’t understand it you know what I mean.”*  Gayle: *“He set up his own doing contracting work, but he had one particular person he did a lot of sub-contracting for who dropped him one summer season without any explanation, and um that hurt a lot, I think he’d got some depression.”*  Peter: *“not really doing anything with any particular purpose…except to keep the day going,”*  **Jack:** *“You don’t want to define yourself by your ailments do you”.*  Peter: *“Not so easy for you though with the [grandchildren} is it?*  **Joan:** *Well I enjoy having them but find I can’t, play with them so much because I can’t get down and get up, I need some hep.* Peter: *Do you find you miss that?* |
| Loss of mobility | Kath: *My daughter got [the walker] for me and I was a bit upset, you know, and I wouldn’t even think about it. But now I know myself mobility is is getting worse,.. I cried…****.****And I just I couldn’t talk to her until the next day and I said to her I’m 72, not 82.*  *Kath: “My balance is very um especially after the fall yesterday I had that shook me up quite a lot actually. I still don’t feel quite right”*  *Gayle: “[H]e did have a pendant alarm with GPS on it which was good…., it gave him a bit of independence…. it worked and it did save him a couple of times. One time he had fallen over and he wasn’t where he said he was going to take the dogs. He was somewhere else entirely but it came up on the tracker and I couldn’t get across the fields to help him but friends did and you know it worked.”*  Peter: *“ [W]e’ve got this armchair… it’s not* ***Joan****’s favourite chair it’s funny, all the various things that we’ve looked to buy that might make the house a bit more comfortable you’re not that keen on are you [laughter]..*  **Joan:** *I’m sat in here all the time, I want to be exercising.*  Peter:*Good point…we [also] bought a chair for the stairs, but I don’t think you [are keen to use it]. Which is good you’re determined to go up and down stairs but of course the worry is having a slip on the stairs would be awful.”* |
| Loss of swallow | *Gayle: “…poor Doug kept getting his nose in the cream, it was very sad, um……Sometimes, he can’t get to the bottom of the cup, um but that’s the problem, he was trying to swallow his pills the other day with just about an inch of drink left in the bottom of the glass and couldn’t get it up to you know….”*  *Gayle: “I was really scared he was going to choke, and I’m not so anxious about that now so.”* |
| Loss of continence | Sue: *“he does a poo but doesn’t know he’s done it, which I’ve no problem, bodily functions don’t phase me at all. I keep him clean, he tries to keep clean, so I said to him I’m going to give you some CosmoCol to sort of pull it together….”*  *Sue: “I find it frustrating, I just said to him I want his quality of life what’s best for him, I don’t mind clearing up, I really don’t mind, but it doesn’t make him happy and very comfortable… [I said] do you want to come shopping, ‘cos we usually go together, and it’s to do with if he makes a mess. I want him to be able to go out and he has incontinence pads which he wears at night but he doesn’t like wearing them during the day, which is understandable..”*  Gayle: “*We had the continence advisor came out about a year ago with a range of techniques, but none of them suited,…. they tried everything else and none of the other stuff, gadgets work, it’s too complicated you know, we can never manage a sheath without a lot of assistance, he didn’t like the feel of it and all of that so, and pads that you stick them on the pants you’ve got to be able to manage getting them off and putting new ones on if you’re going to have any independence and self respect, so it’s pull up pants….that’s eased it a bit.”*  Gayle: “*I might sound cowardly but I’m frightened, I’d be worried that we’d have an incident like we had on the motorway last Summer. There was an accident and a very long hold up, we were stationary for about an hour. He’d got his paddy pants on, but he insisted on getting out of the car. and he’s trying to open the door, and in the end we tried tearing up another pear of padded pants and stuffing them down his trousers and that didn’t help, we couldn’t do it, and we hadn’t got a bottle with us then”.*  Gayle: *“Just I get more and more exasperated. Around the situation you know, um getting a bit, we just sit down and just start a meal and he pops out to the loo or something”.*  Linda: *“He struggled* *‘cos they’ve changed his medication, he’s on medication for depression, anxiety, which gave him diarrhoea, he couldn’t go out anywhere”* |
| Loss of independence/freedom | Person with Lewy body dementia:  **Jack**: *“I mean I think we, Linda more than me, are pretty good at socialising, considering that I haven’t got a car, I can’t drive.”*  **Jack** and Linda decided to move *“because it was out of the way, there’s no pavements or anything and Jack doesn’t drive.”*  **Jack** says not being able to drive *“is a real bummer”.* **Jack:** *“I gave up voluntarily ‘cos I could see the way it was going, and I thought the thing is to get used to.* *Well I realised I’d got, or it was highly likely that I had this [Lewy body dementia].* *Linda:* *It’s the hallucinations that’s the issue, if you’re driving a car it’s seeing [things].”*  **Patrick:** “*…. The bottom line about that is that er it’s not necessarily your vision being changed, the thing that’s more dangerous is you might have a black out. You can’t predict it. So that’s what stops you, and I really couldn’t go out and kill a few people. But it’s the vision you know, it’s a serious thing. And it does happen apparently.”*  Gayle: *Yeah that was the last time you [drove] wasn’t it….***Doug:** *Sadly I can’t do it anymore”.*  Sue: *“he’s not quite as confident and I don’t know whether I’m overhelping him because I don’t want to him to lose his confidence or his independence um but.”*  **Patrick:** “*there’s a certain sort of sense of er being pinched in a bit, but they’re looking after me.* Interviewer: *How do you mean pinched in a bit?* **Patrick:** *Just constricted. Not like I’m free like a bird….Um, there the situation I’m in is, can’t think of the word…..I’m not locked up but there is a certain constrictment, getting around.… but I can’t go out and go driving or anything like that”.*  **Joan***:” I was capable of being able to walk, whereas now I’m mostly being prisoner here. Interviewer: Prisoner here, you mean in bed?*  **Joan:** *Mmm. I have all my um meals… It’s frustrating…Unfortunately she [the occupational therapist] concluded that I wasn’t ready to be released.”*  **Kath: “***I do fight it, very much so, ‘cos I won’t let it get hold of me”.*  *Um mobility at the moment is quite frustrating for me now. I do have a walker. My daughter got it for me and I was a bit upset, you know, and I wouldn’t even think about it. But now I know myself mobility is getting worse, so you know…I cried….”*  Spousal Caregiver:  Sue: “*I just cannot plan anything, I mean a group of friends went to the cinema last week, I can’t you know I can’t do it, not during the week,”*  Gayle: *“I like to contribute to the village and I can’t you know as much…., so I don’t get the social contact that I used to. Um I just can’t do all the things I like to do,”*  Gayle: *“Constricting now, because he needs somebody here, I might nip down to the shop and back, about half an hour, but um ……you know all the time you’re having to think about the cover. I’ve got friends in the village who very kindly offer to help, but because of the loo business, not everybody you can ask to do that”.*  Gayle: *“it does constrain my lifestyle and I know his lifestyle’s not really….”* |
| Being a couple/anticipated future | Peter:.…*the thing I miss most is it’s difficult to do things together as we would have done before, to get out would be lovely I mean I can but obviously Joan can’t but um I think that’s what we miss most isn’t it, just to go places and do things together*.”  Gayle: *“it does constrain my lifestyle and I know his lifestyle’s not really but I thought I was going to have a retirement with old tractors on the drive being repaired, ‘cos he and a friend used to do up some vintage tractors a few years back….”*  Gayle: *“Um it’s quite sad really ‘cos it’s mostly the women who make an effort to go and chat……I think the men just don’t know what to talk about”.*  Ken: “*I looked after you all year, you don’t need to have anything special on your birthday.*  **Kath:** *“I find that when things get difficult between Ken and me...I go out into the garden and that has become my retreat if you like going out there”.* |
|  |  |
| Loss of companionship/person/shared sense of reality | Peter: “*I suppose the other things I miss most is just the companionship really, that’s hardest because we’re not sharing anything in the sense of other than me putting a spoon in her mouth, that’s it now really.”*  Peter: *“I think the other thing you get this guilt. I suppose it’s guilt, you feel I should be, ‘cos I don’t feel I’ve ever I’m not really doing anything with any particular purpose, does that make sense, the purpose is keeping the day going but not actually doing anything of any substance really,*”  *“Probably sadly the most I ever talk to* ***Joan*** *about are some of those things which aren’t real anyway, you know the discussions we have are about this boy called Johnny, who we don’t know, but in* ***Joan****’s mind Johnny comes up every now and again...”*  Peter: “*[I]t’s again adjusting to our relationship because I said to some people when they’d asked me, it feels like we’ve in a way been divorced, but not, but still living together, because it’s a different feeling between us isn’t it, which is not your fault at all… . I miss what we had, a lot [laughter]. I don’t think it necessarily has quite the same impact in* ***Joan****.”*  Sue: “*I think the thing is* ***Patrick’s*** *personality is there but it um you know it’s I want to say it’s changed,…”*  Sue: “*[Sometimes] I’m a Cook. I was the gardener yesterday.…* *At the beginning it was really quite distressing, um but er you know I will sit and he’ll suddenly say where’s the nice lady that made the tea.*  **Patrick:** *I can’t explain that.”*  Sue: …. “*I mean it’s the most horrendous um I was going to say medical condition. My mother had cancer, Patrick’s mum had dementia and we both decided that having cancer was better than having dementia. Although my mother knew she was very ill, she knew what was happening, whereas sometimes he doesn’t*.”  Ken: “*[S]he kept shouting*  *out I was trying to murder her and what not and there was people in the house who would walk from one room to another.* **Kath:** *I’ve got no recollection honestly of any of it.”*  **Jack: “***I mean that’s great fun ‘cos we sort of have um a meeting and the women will make something to eat, you know and ….. [it’s the] companionship, that sounds a bit weak…. sounds more about like wearing slippers a bit but er well it’s important to me.”*  **Jack:** *[The hallucinations] were so realistic… I assumed it was real. But Linda said no they’re not, who has the right of it, I don’t know. Linda: Me [laughter].*  **Jack:** *Well that’s inevitable, I’ve lost already…*. *Yeah I mean you know you could get two very different answers depending in whether it’s me or Linda that’s being interviewed.”*  *Linda: “They were quite scary images weren’t they.* **Jack:** *They were scary and um yeah but we did get over it and things gradually improved”.*  *Gayle: “if you’re long periods with somebody who can’t be left on their own and they’re asleep it gets quite lonely,”* |
